# Impact of predisposing, enabling, and need factors in accessing preventive medical care among U.S. children: results of the national survey of children's health

**DOI:** 10.1186/1750-4732-2-12

**Published:** 2008-12-08

**Authors:** Ka-Ming Lo, Kimberly G Fulda

**Affiliations:** 1 Biostatistics Department, University of North Texas Health Science Center, 3500 Camp Bowie Blvd, Fort Worth, TX 76107, USA; 2 Primary Care Research Institute, Department of Family Medicine and Community Medicine, University of North Texas Health Science Center, 855 Montgomery, Fort Worth, TX 76107, USA

## Abstract

**Background:**

Preventive care in the United States has been a priority, especially for children under 18 years of age. The objective of this analysis was to determine which predisposing, enabling, and need factors affect access to preventive health care for children.

**Methods:**

Data were obtained from the National Survey of Children's Health (NSCH), a cross-sectional study of children in the United States. The current analysis examined whether predisposing, enabling, and need factors included in Andersen's Socio-Behavioral Model significantly affect having received preventive medical care among children 3–17 years of age. Logistic regression was used to compute odds ratios and 95% confidence intervals.

**Results:**

63,924 out of 85,151 subjects were reported as having received preventive medical care. After stratifying by geographical region, the following factors were significant for predicting having received preventive care. Age was negatively associated with having received care in all four regions. Household education of less than a college degree and being white (compared to black) were negatively associated with having received care in the Northeast, Midwest, and South. Having fewer than 4 children was negatively associated in Northeast but positively associated in the West with having received care. Being male, having less than 3 children in the household, having less than 3 adults in the household, and being Hispanic were positively associated with having received care in the West only. Not having insurance and having a lower socioeconomic status were negatively associated with having received care; while, having a personal doctor or nurse was positively associated in all four regions. Primary language other than English was negatively associated with having received care in the Northeast only. Currently needing medicine was also positively associated with having received care in all four regions; while, having limited abilities to do things was positively associated in the West only.

**Conclusion:**

Older children whose family resides in Northeast, Midwest, and South regions with low household education and poverty levels experience insufficient preventive health care. Medicaid or SCHIP coverage should be expanded for children who are still uninsured. For children in the West, gender, family size, ethnicity, and their ability to do things should also be considered when providing assistance for receiving preventive care.

## Background

In 1997, the American Academy of Pediatrics (AAP) Committee on Child Health Finance set forth 22 services that individuals up to age 21 should be able to access for "optimal health and well-being". The recommended services include medical care such as "health supervision with preventive care and immunizations according to the AAP's 'Recommendations for Preventive Pediatric Health Care"' [1]. Routine visits to a pediatrician provide the opportunity for preventive care through well child examinations and family centered care. The AAP recommends 28 well child visits between birth and 21 years of age. Beginning at age 3, one yearly routine preventive care visit is recommended [1,2].

The Andersen's Socio-Behavioral Model is a widely used model in research on use of health care services [3-7]. Its initial model was developed in the 1960s [8], and through time it has been expanded and modified. Modern study of health care use and access has shifted from an individual level focus to a combination of the individual, the health care system, the external environment, and the effects that each have on the others. The Andersen model [8] applied in this study examined 3 determinants of health care use in children: predisposing factors, enabling factors, and perceived needs.

Predisposing factors include biological factors that may influence the likelihood an individual needs a health service, social structure that may influence how an individual can cope with health problems, and health beliefs that may influence an individual's perception of their need for a health service [8]. Predisposing factors include demographic characteristics and socio-structural characteristics such as education level, race and ethnicity, and family size. Previous research has demonstrated mixed results about the effect of race and ethnicity and family size on the use of pediatric preventive services [6,9-12]. A higher maternal education level, however, is associated with an approximate two times increase in the likelihood of having received a routine visit in the past year for children [6].

Enabling factors, or resources, include family characteristics such as income, insurance coverage, access to services (transportation and distance to care), and community characteristics such as availability of resources and region of the country. Low family income, being uninsured, and having a regular clinician have been identified as risk factors for inadequate access to preventive health care [10,12]. Primary language (English versus non-English) and geographical region of the country have also been found to be associated with access to care [13]. For example, Spanish speaking Hispanics report decreased odds of having a physician visit, a mental health visit, a mammogram, and an influenza vaccination in the last year after controlling for predisposing, enabling, and need factors as compared to English speaking Hispanics [14]. For geographical region, living in the West is associated with not being satisfied with child health care [1].

Perceived needs refer to the revised Andersen model and account for the subjects' health beliefs or psychosocial factors [15] when measuring access to health care services. Perceived needs may include aspects of the subject's attitudes, values, and knowledge about health problems and services that affect their perception of whether they do or do not need health services. Hughes and Wingard found that parental beliefs, specifically about the timing of routine visits, were associated with having received preventive care in the past year [6]. Parents of children were asked how often they felt their child should see a doctor or health care professional. If the parent's answer matched the AAP's guidelines, the child was 2.8 times more likely to have had a routine visit in the last year as compared to children of parents whose response did not match the AAP's guidelines [6].

One critique of the Andersen model is a lack of definition of access in the original model [16]. According to Andersen, four types of access are defined using multidimensional terms through different aspects of later versions of the behavioral model as used in the current analysis. Potential access refers to the existence of resources which is measured by enabling factors. Andersen suggested that more enabling resources equates to a greater use of health services. Realized access is defined as the use of health services. Equitable access depends on demographic characteristics and need factors, while social structure and health beliefs as described in predisposing factors and enabling resources are responsible for inequitable access [8].

Most of the studies regarding the use of preventive health care for children have focused on insurance, income, education, and differences among racial/ethnic groups. Andersen's work suggests that other predisposing, enabling, and need factors exist and also play a role in influencing access to health care. In this study, the main goal was to examine which of these predisposing, enabling, and need factors affect access to preventive health care for children.

## Methods

### Data source

This study included secondary data analysis of data obtained from the 2003 National Survey of Children's Health (NSCH), a national survey conducted by the Centers for Disease Control and Prevention's National Center for Health Statistics. The NSCH utilizes the State and Local Area Integrated Telephone Survey (SLAITS), which is a continuous surveillance system used to monitor the health of U.S. citizens at state and local levels. SLAITS utilizes random digit dialing to obtain a random sample within 78 primary sampling units every three months. The primary sampling units are self contained and do not cross state lines, allowing for analysis by geographical region.

The NSCH contains a variety of questions addressing children's physical and mental health. Data are collected from an adult member within the household who has the greatest knowledge about the selected child's health. The NSCH 2003 dataset included 102,353 children up to 17 years of age. Guidelines from the AAP state that children less than three years of age are required to have more than one preventive care visit each year, whereas, children three years of age and older should have one preventive care visit each year. NSCH questions allow for the determination of having a preventive care visit in the past 12 months. Therefore, only children ages 3 to 17 years of age were included in this study, resulting in a final sample size of 85,151 subjects for the current analysis. Study procedures for this analysis were approved by the University of North Texas Health Science Center Institutional Review Board.

### Dependent variable

Children were identified as having received care in the past 12 months if a response of "Yes" was provided to the following question "During the past 12 months, did [CHILD] see a doctor, nurse, or other health care professional for any kind of medical care, including sick-child care, well-child check-ups, physical exams, and hospitalizations?" and the respondent reported at least 1 visit for the following question "During the past 12 months, how many times did [CHILD] see a doctor, nurse, or other health care professional for preventive medical care such as a physical exam or well-child check-up?"

### Independent variables

Following Andersen's Socio-Behavioral Model, seven variables were identified as predisposing factors including age, gender, number of adults in the household, number of children in the household, highest education level of the household (less than high school, high school graduate, at least some college), race (white, black, multiple races, other), and ethnicity (Hispanic, non-Hispanic). Five variables were identified as enabling factors including primary language spoken in the household (English, other), insurance status (continuously covered by any insurance over the last 12 months or not), poverty level (< 100%, 100 – 199%, 200 – 299%, 300 – 399%, ≥ 400% of the federal poverty level), having a personal doctor or nurse (yes, no), and geographical region of the household (Northeast, Midwest, South, West). Two variables were identified as need factors including whether the child currently needed or used medicine other than vitamins (yes, no) and whether the child was limited or prevented in any way in his/her ability to do things most children of the same age do (yes, no).

### Statistical analyses

All analyses were conducted using SPSS with complex samples module to account for complex survey weighting and the sample design. The overall descriptive statistics are presented in Additional file 1. Simple logistic regression was performed for each predictor variable. Multiple logistic regression was used to predict having received preventive health care in the past 12 months while controlling for all potential risk factors. Potential first degree interactions were tested based on previous literature. The ethnicity × geographic region interaction was significant. Therefore, multiple logistic regression was performed for each geographic region individually. Unadjusted and adjusted odds ratios and 95% confidence intervals are presented in Additional file 2 and Additional file 3. Analyses were considered statistically significant at the alpha 0.05 level.

## Results

Of the 85,151 respondents, a total of 75.1% (63,924) children were reported as having received preventive medical care during the last 12 months (Additional file 1). The corresponding rates of receiving adequate care among the 4 regions were: 86.5% in Northeast, 75.0% in Midwest, 74.0% in South, and 68.9% in West (Additional file 1). Compared to the Northeast, unadjusted odds ratios revealed less chance for children to have received preventive medical care by 53.5% in the Midwest, 55.7% in the South, and 65.5% in the West (Additional file 2). After stratifying by geographic region, risk factors for having received care in the past 12 months varied.

### Northeast

#### Predisposing factors

Every year increase in age was associated with a 5 percent decrease in the odds of having received preventive care in the past 12 months (OR: 0.95; 95% CI: 0.93–0.97). Additionally, households with 1 child were 33 percent less likely to have received care (OR: 0.68; 95% CI: 0.47–0.97) as compared to households with 4 or more children, and having a highest household education of less than high school (OR: 0.54; 95% CI: 0.31–0.96) or a high school degree (OR: 0.74; 95% CI: 0.59–0.92) decreased the odds of having received care as compared to having a college education. Black children, however, were 43 percent more likely to have received care (OR: 1.43; 95% CI: 1.03–1.99) as compared to white children.

#### Enabling factors

Children from households with a primary language other than English (OR: 0.52; 95% CI: 0.32–0.84) were 48 percent less likely to have received care. Additionally, children who were not continuously covered by insurance over the last year (OR: 0.63; 95% CI: 0.48–0.83) were 37 percent less likely to have received care. Poverty level also significantly affected having received preventive care in the last 12 months. Compared to households at or above 400% of the federal poverty level (FPL), households at < 100 of the FPL (OR: 0.45; 95% CI: 0.31–0.67), 100 – 199% of the FPL (OR: 0.49; 95% CI: 0.36–0.65), 200 – 299% of the FPL (OR: 0.58; 95% CI: 0.45–0.74), and 300 – 399% of the FPL (OR: 0.73; 95% CI: 0.56–0.94) were less likely to have received care. Having a personal doctor or nurse doubled the odds (OR: 2.00; 95% CI: 1.51–2.65) of a child having received preventive care.

#### Need Factors

Children who currently need or use medicine were over 2 times more likely (OR: 2.28; 95% CI: 1.72–3.02) to have received preventive care.

### Midwest

#### Predisposing factors

Every year increase in age was associated with a 4 percent decrease in the odds of having received preventive care (OR: 0.96; 95% CI: 0.95–0.97). Compared to households with a highest education of at least some college, having less than a high school degree (OR: 0.65; 95% CI: 0.46–0.91) or a high school degree (OR: 0.73; 95% CI: 0.65–0.83) was associated with decreased odds of having received care. Black children were 46 percent more likely (OR: 1.46; 95% CI: 1.19–1.78) than white children to have had preventive care.

#### Enabling factors

Children who were not continuously covered by insurance the past 12 months had an 18 percent decreased likelihood of having received preventive care (OR: 0.82; 95% CI: 0.70–0.95) compared to insured children. Households with a lower poverty level had decreased odds of having received preventive care compared to households at or above 400% of the FPL: < 100% of the FPL (OR: 0.77; 95% CI: 0.62–0.94); 100 – 199% of the FPL (OR: 0.72; 95% CI: 0.620–0.84), 200 – 299% of the FPL (OR: 0.70; 95% CI: 0.61–0.80), 300 – 399% of the FPL (OR: 0.81; 95% CI: 0.71–0.93). Having a personal doctor or nurse increased the odds (OR: 1.56; 95% CI: 1.35–1.81) of a child having received preventive care by over 50 percent.

#### Need Factors

Children who currently need medicine had over two times the odds (OR: 2.34; 95% CI: 1.04–2.67) of having received care.

### South

#### Predisposing factors

A one year increase in age was associated with a 5 percent decrease in having received care (OR: 0.95; 95% CI: 0.94–0.96). Compared to having a highest household education of at least some college, children from families with a highest household education of less than high school (OR: 0.77; 95% CI: 0.60–0.99) or high school degree (OR: 0.81; 95% CI: 0.72–0.91) were less likely to have received preventive care in the past 12 months. Black children (OR: 1.42; 95% CI: 1.25–1.62) and children from multiple races (OR: 1.37; 95% CI: 1.00–1.88) were 42 percent and 37 percent more likely than white children to have received preventive care, respectively.

#### Enabling factors

Children who were not continuously covered by insurance the past 12 months had a 34 percent decreased odds (OR: 0.66; 95% CI: 0.57–0.76) of having received preventive care than insured children. Additionally, compared to households at or above 400% of the FPL, households at 100 – 199% of the FPL (OR: 0.68; 95% CI: 0.59–0.80), 200 – 299% of the FPL (OR: 0.71; 95% CI: 0.61–0.82), and 300 – 399% of the FPL (OR: 0.77; 95% CI: 0.67–0.89) were less likely to have received care. Having a personal doctor or nurse doubled the odds (OR: 2.09; 95% CI: 1.84–2.38) of a child to have received preventive care.

#### Need Factors

Children who currently need medicine had a higher odds (OR: 2.39; 95% CI: 2.09–2.73) of having received care.

### West

#### Predisposing factors

Every year increase in age was associated with a 7 percent decrease in the likelihood of having received preventive care in the last 12 months (OR: 0.93; 95% CI: 0.91–0.95); while, male children were 22 percent more likely than female children to have received preventive care (OR: 1.22; 95% CI: 1.04–1.43). Unlike the other geographic regions, the total number of children and adults in the household was associated with having received preventive care in the past 12 months. Children from families who had 1 child (OR: 1.53; 95% CI: 1.17–2.00) or 2 children (OR: 1.38; 95% CI: 1.06–1.78) were more likely to have received care as compared to families with 4 or more children. Children from families with 1 adult (OR: 1.60; 95% CI: 1.21–2.11) or 2 adults (OR: 1.38; 95% CI: 1.12–1.71) were also more likely than families with 3 or more adults to have received preventive care. Additionally, Hispanic children had a 1.5 times higher odds (OR: 1.55; 95% CI: 1.18–2.02) than non-Hispanic children to have had preventive care.

#### Enabling factors

Children who did not have continuous insurance coverage during the past 12 months had a 29 percent lower odds (OR: 0.71; 95% CI: 0.58–0.87) of having received care than those who were continuously covered. Children from households with lower poverty levels had decreased odds of having received preventive care compared to children from households at or above 400% of the FPL: < 100% of the FPL (OR: 0.70; 95% CI: 0.51–0.98); 100 – 199% of the FPL (OR: 0.74; 95% CI: 0.58–0.96), 200 – 299% of the FPL (OR: 0.72; 95% CI: 0.57–0.91). Having a personal doctor or nurse doubled the odds (OR: 2.04; 95% CI: 1.67–2.51) of a child having received preventive care.

#### Need factors

Children who currently need medicine were over two times more likely (OR: 2.02; 95% CI: 1.59–2.57) to have received preventive care in the last 12 months. Additionally, children who had limited abilities to do things were also more likely (OR: 1.48; 95% CI: 1.00–2.20) to have received preventive care.

Detailed results in each geographical region are presented in Additional file 3.

## Discussion

Among the 13 predisposing, enabling, and need factors included in the model, nine factors in the Northeast, seven factors in the Midwest and the South, and 10 factors in the West were significant for predicting having received health care in the past 12 months for children 3 – 17 years of age. Previous literature suggests a higher risk of experiencing unmet health care needs in the West for children with special health care needs [17]. The current study provides further insight into factors associated with having received preventive care in the past 12 months. Additionally, the current analysis delineates factors associated with having received care by geographical region of the household. This stratification has not been presented elsewhere in the literature and contributes to the pool of knowledge necessary to adequately address access to routine preventive medical care, one of the basic necessities for ensuring a healthy childhood.

Differences in risk factors within regions were identified. Specifically, factors associated with having received preventive care in the past 12 months were different in the West as compared to the other regions. In the West, if a child was uninsured, non-Hispanic, female, from a large family, from a household with a poverty level below 300% of the FPL, did not have a personal doctor or nurse, did not need medicine, or was limited in his/her ability to do things, the child was less likely to have received preventive medical care in the last 12 months.

Risk factors that varied between the West and the other geographic regions included gender, number of children in the household, number of adults in the household, highest household education, ethnicity, race, and having limited abilities. These differences can impact programs and policies aimed at increasing preventive care among children. For example, gender is only significant in the West, demonstrating higher odds of receiving care in males than in females. This is counterintuitive when compared to a previous study [17] that found males were more likely to not have a usual source of care than females. Gender was not significant in the other regions. Additionally, Hispanic children in the West were 1.55 times more likely than non-Hispanic children to have received preventive medical care. Children in the West who were limited in their ability to do things were also more likely to have received preventive medical care, but this association did not hold true for the other regions.

Whereas race was not significantly associated with having received preventive care in the West, it was in the other three regions. In the Northeast, Midwest, and South, black children were more than 40% more likely to have received preventive care in the past 12 months. This disagreed with the findings in Shi's study which stated that blacks were less likely to have a usual source of care compared to whites [18]. Children of multiple races were also more likely than white children to have received care in the South. Insurance status and poverty level were both associated with having received preventive medical care in the past 12 months among all geographic regions. Some previous studies had also found similar results [19-21].

Primary language is only significant in the Northeast region. Participants who did not speak English as their primary language experienced 48% lower odds in accessing preventive care, which agreed with Woloshin's suggestion [13] of association between language and using preventive services. This finding suggests the presence of communication barriers in the Northeast region. No associations, however, were identified in the other regions.

For all geographic regions, having a personal doctor or nurse was associated with a 1.5 times increase (Midwest) or 2 times increase (Northeast, South, and West) in the likelihood of having received preventive care in the past 12 months. While this seems intuitive, it somewhat contradicts recent research published by Inkelas et al [10]. Inkelas et al. found that children with a regular clinician were 1.8 times more likely to experience delayed preventive care as compared to children without a regular clinician. Inkelas et al., however, did not control for socioeconomic status but did control for well child care setting. Results from Inkelas et al. discuss receiving delayed care, while the current analysis presents findings on having received care in the past 12 months [10]. The impact of having a personal doctor or nurse on receiving care should be explored further.

## Limitations

There are several potential limitations to this analysis. First, the data represent information collected from a cross-sectional study of children's health. As such, no conclusions about temporal associations can be made. Additionally, data were collected from a person in the child's household, not through medical records review. This leaves a potential for recall bias. Results should also be interpreted carefully since the magnitude of the odds ratios cannot be compared across strata due to potential error among the models. Finally, the current analysis only included children three years of age and older and may not apply to younger children.

## Conclusion

Generally, within the Northeast, Midwest, and South regions, programs to increase the use of preventive care should target white, multiple race, and other race with a lower household education level and household income below 400 percent of the federal poverty level. Additionally, programs such as Medicaid and SCHIP should focus on uninsured children in these regions. In the West region, preventive care programs should target female children, households with a relatively large family size, and children who have limited abilities. Non-Hispanic children are also experiencing inadequate preventive care in the West. Finally, risk factors specific to a geographic region should be considered to adequately improve the use of preventive care by children.

## Abbreviations

SCHIP: State Children's Health Insurance Program; AAP: American Association of Pediatrics; NSCH: National Survey of Children's Health; SLAITS: State and Local Area Integrated Telephone Survey; FPL: Federal Poverty Level; OR: Odds Ratio; CI: Confidence Interval.

## Competing interests

The authors declare that they have no competing interests.

## Authors' contributions

KL was involved in the literature review, algorithm development, data analyses, results interpretation, and manuscript preparation. KGF was involved in the literature review, algorithm development, results interpretation, and revising the final draft. All authors have approved the final draft.

## Supplementary Material

Additional file 1 Table 1. Overall demographic characteristics. Click here for file

Additional file 2 Table 2. Unadjusted risk factors for having received preventive care. Click here for file

Additional file 3 Table 3. Adjusted risk factors for having received preventive care stratified by geographic region. Click here for file
